# Region sampling NeRF-SLAM based on Kolmogorov-Arnold network

**DOI:** 10.1371/journal.pone.0325024

**Published:** 2025-05-27

**Authors:** Zhanrong Li, Jiajie Han, Chao Jiang, Haosheng Su

**Affiliations:** 1 School of Computer and Electronic Information, Guangxi University, NanNing, China; 2 Nanning Huishi Technology Co., Ltd., NanNing, China; 3 Guangxi Intelligent Digital Services Research Center of Engineering Technology, Nanning, China; 4 Key Laboratory of Parallel, Distributed and Intelligent Computing(Guangxi University), Education De-partment of Guangxi Zhuang Autonomous Region, Nanning, China; Beijing University of Technology, CHINA

## Abstract

Currently, NeRF-based SLAM is rapidly developing in reconstructing and bitwise estimating indoor scenes. Compared with traditional SLAM, the advantage of the NeRF-based approach is that the error returns to the pixel itself, the optimization process is WYSIWYG, and it can also be differentiated for map representation. Still, it is limited by its MLP-based implicit representation to scale to larger and more complex environments. Inspired by the quadtree in ORB-SLAM2 and the recently proposed Kolmogorov-Arnold network, our approach replaces the MLP with a KAN network based on Gaussian functions, combines quadtree-based regional pixel sampling and random sampling, delineates the scene by voxels, and supports dynamic scaling to realize a high-fidelity reconstruction of large scenes for a SLAM system. Exposure compensation and VIT loss are also introduced to alleviate the necessity of NeRF on dense coverage, which significantly improves the ability to reconstruct sparse outdoor view environments stable. Experiments on three different types of datasets show that our approach reduces the trajectory error accuracy of indoor datasets from centimeter-level to millimeter-level compared to existing NeRF-based SLAM and achieves stable reconstruction in complex outdoor environments, considering the performance while ensuring efficiency.

## Introduction

Simultaneous localization and mapping (SLAM) [1] is widely used in UAVs, autonomous driving, and mixed reality to help robots autonomously perform localization, mapping, and path planning functions without human control. Its goal is to construct dense or sparse maps of unknown environments while tracking camera poses. Traditional SLAM methods utilize point clouds, meshes, voxels, etc as scene representations to construct dense maps and employ feature points [2–5], optical flow [6,7] or direct methods to estimate camera poses, and ultimately combine the estimated poses with the corresponding scene representations to update and optimize the global map. Although these methods have been continuously investigated for a more extended period and have shown good reconstruction results, they have not achieved better results in presenting new views, estimating unobserved regions, etc.

With the advent of Neural Radiation Field (NeRF) [8], for a given unknown camera viewpoint, its rendering method of using a multilayer perceptron (MLP) to map the query 3D points to occupancy or color, and ultimately synthetically rendering out the view from that viewpoint has attracted a lot of attention. Compelled by the fact that training NeRF requires known camera poses and corresponding views, while camera poses are unknown quantities in SLAM applications, a growing body of work [9–11] applies NeRF’s techniques to estimate camera poses and simultaneously model the environment, i.e., NeRF-based SLAM. This aspect of research is gradually showing advantages in generating high-quality, dense maps with low memory consumption.

The advantages of NeRF-based SLAM are the unified framework, high reconstruction quality and the ability to handle unknown regions, but the problems of high computational resource consumption, limited real-time and generalization ability, and extensive model network forgetting limit the development of the technology. At this stage of research, simplifying the neural network structure and improving the sampling strategy are usually used to reduce the problem of excessive resource consumption during the computation process, such as Go-SLAM [12] generates total sampling points along the ray during rendering, where some of the points use hierarchical sampling and the other points are selected near the depth value. Improvements for generalization capability such as RO-MAP [13], vMAP [14] train a separate MLP for each target to build object-level NeRF maps, and LiDAR-NeRF [15] improves the model’s ability to sense the environment and generalize by introducing a variety of information such as LiDAR point clouds.The MLP network structure has been shown to suffer from catastrophic forgetting, which tends to learn new information while learning new information quickly and drastically forgets previously learned information. To address the extensive range of scenarios in existing SLAM datasets, solving the MLP large-scene network forgetting problem is one of the focuses of NeRF-based SLAM. Recent works such as Vox-Fusion [16], iMap [11] and its subsequent works [17,18] have used neural networks combined with voxel grids and feature grids to achieve reconstruction and position estimation of indoor scene datasets more excellently. There exist a large number of urban road scene datasets in SLAM-related datasets, such as KITTI [19], Waymo-Open-Dataset [20] and Apollo [21]. Existing work has shown poor generalization in the training process of the above dataset, and the interference of many unfavorable factors, such as light transformations, dynamic objects, etc., needs to be considered. Since the camera coverage of the urban road dataset is sparse compared to the NeRF dataset, the effect of sparse views also needs to be considered when designing the SLAM system.

To address the above issues, recent work constrains the generated results by introducing regularizations such as spatial viewpoint [22], frequency, and occlusion [23] during the training process to reduce artifacts and distortions due to sparse views, and to improve the realism and accuracy of the rendering results. Co-SLAM [24] violently maintains all frames as keyframes, which are learned by multiple training repetitions during mapping, to alleviate the problem of network forgetfulness. It is proposed in Kolmogorov-Arnold Networks [25] that KAN networks can circumvent the problem of network forgetting at the source by placing the activation function on the weights without being prone to catastrophic forgetting like MLPs. For the problem of outdoor scene illumination, a generative potential optimization method [26,27] was proposed to achieve illumination consistency by assigning an appearance embedding vector of corresponding length to each image. At the same time, by changing the parameters of the appearance embedding vectors to change the scene’s lighting conditions, the model can be allowed to learn the characteristics of the scene and the law of change in various situations and enhance the generalization of different scenes. In addition, the problem of low localization accuracy of NeRF-based SLAM is also proposed. Go-SLAM [12] optimizes the bit position by introducing BA and loopback to improve localization accuracy. NeRF-Loc [28] combines the descriptors of the traditional SLAM and uses the learned conditional NeRF 3D model to compute 3D descriptors to directly match with the images to achieve coarse to fine visual localization.

This paper will use neural implicit networks and voxels to reconstruct and estimate positions more accurately for indoor and urban road datasets. Inspired by the successful application of voxels and neural implicit expressions [16] in parallel tracking and map building, we propose a new hybrid data structure that combines voxels and KAN networks [25]. Specifically, we use voxels as the basis for map building, i.e., the underlying map of the scene is represented as a collection of voxels, and the KAN network is embedded as a color query module, together with regionally chromatically sampled pixels to mitigate the network catastrophic forgetting problem of the network model in large scenes. Some diffusion model-based approaches [29,30] demonstrate that a small number of data can generate viewpoint consistency, and we introduce semantic pseudo-labeling to guide the training, ensuring good quality of local texture perception and global structure refinement under sparse views. For the problem of light transformations in outdoor scenes, we propose an exposure compensation model based on the white balance algorithm [31], which assigns a certain ratio of original white points to each image as embedding vectors to train the model as a way to achieve light consistency. Our experiments show that high-fidelity reconstruction of indoor scenes and stable reconstruction and position estimation of urban street scene datasets can be achieved by integrating multiple techniques.

This paper is organized as follows: a review of related work is given in Section Related Work; We explain our approach and methodology in Section Methods; In Section Results, we evaluate the proposed system on three different datasets and present data to argue our conclusions. Finally, we conclude our paper by summarizing in Section Discussion.

## Related work

### Large-scale scenes

Due to the limited model capacity, purely MLP-based neural radiation fields often need to be more balanced and present ambiguous rendering on large-scale scenes. Block-NeRF [32] chooses to decompose a large scene into multiple independently-trained NeRFs. This decomposition decouples the rendering time from the size of the scene, and the rendering can be scaled up to arbitrarily large environments and allows for each block of the environment to be updated. Mega-NeRF [33] decomposes the scene into units with prime points and initializes the corresponding model weights, with each weight submodule being a series of fully connected layers similar to the NeRF [8] architecture.

iMap [11] verifies that NeRF can perform good void completion in SLAM map building, which lays a solid foundation for NeRF-based SLAM and also verifies that MLP can be used as a unique scene representation for RGBD-SLAM but suffers from the problem of network forgetting for large scenes. Its follow-up work, Nice-SLAM [17], realizes large-scale indoor scene reconstruction by introducing a hierarchical scene representation based on feature grids, combining multi-resolution spatial grids using multiple MLPs, and using pre-trained geometric priors. While this approach solves the problem of network forgetting for large scenes, using multiple MLPs results in the computation time being too long. Vox-Fusion [16] takes an alternative approach by combining NeRF with sparse voxel grids using octrees combined with Morton codes to achieve fast voxel assignment and retrieval. It also supports dynamic scaling of the scene, which means that instead of predefining the map size like NeRF, the map can be built incrementally like SLAM.

### Sparse view

Regularization methods can be used in neural radiation fields [34] to optimize the performance and generalization ability of the model and constrain the generated results by introducing a corresponding loss function during training to achieve the desired results. RegNeRF [22] proposed geometric regularization and appearance regularization, which can be used to improve the synthetic view by constraining the spatial continuity of the scene and constraining the consistency of the model across different viewpoints during training to enhance the quality and accuracy, reducing artifacts and distortions in the views. FreeNeRF [23] introduces free frequency regularization, which allows the frequency range to vary freely during training to accommodate different samples, which provides a better balance between high-frequency details and the model’s generalization ability.

PixelNeRF [29] proposes that the original NeRF fails to utilize the known information fully. It takes views from known perspectives, extracts image features by pre-training ResNet, and uses the features as supplementary inputs for neural rendering. DietNeRF [30] and CLIP-NeRF [35] argue that it is easy for a human to detect from the semantic cues whether the two images are of the same object in a view and therefore proposed semantic consistency loss. Specifically, CLIP VIT extracts the rendered semantic representations and then maximizes the similarity with the true-value view representations. Sin-NeRF [36], contrary to the above, argues that the densely-covered viewpoints required by NeRF limit the development and attempts to train the radial field on realistic and complex scenes using single viewpoints by using designed semantic and geometric canonicals. However, all the above methods are taught using 2D features as constraints and need to introduce actual a priori knowledge, making their reliability questionable. MVSNeRF [37] utilizes planar skimming cost volumes (widely used in multiview stereo) for geometric-aware scene inference and combines it with physically-based volume rendering for neural radiation field reconstruction. GeoNeRF [38] utilizes the Transformer to render and infer geometry and appearance while utilizing voxel rendering to capture image details, resulting in a new viewpoint synthesis method based on NeRF [8] for generalized realism.

### Outdoor environment

NeRF-W [26] enhanced rendering with additional transients and appearances as inputs to better account for illumination differences and transient occlusions between images while proposing the generation of a latent optimization method [39] that assigns an appearance embedding vector of the corresponding length to each image to achieve illumination consistency. Urben-NeRF [27] proposed that the sky, an infinitely far away element of the outdoor environment, would affect the solid structure, introducing image prediction segmentation to supervise the density of rays pointing towards the sky.

## Methods

**Fig 1:**
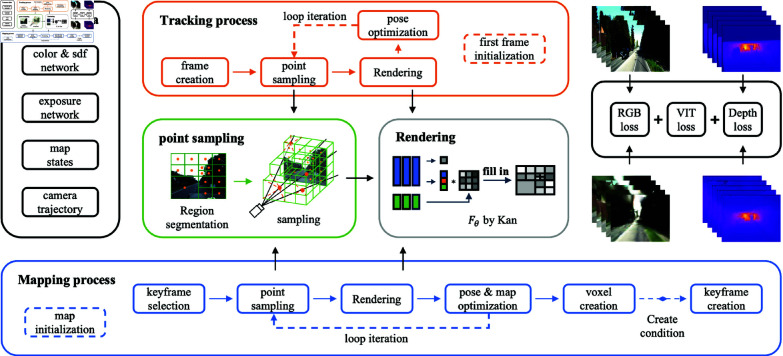
Overview of our system. Our system consists of four parts: (1) region sampling, which samples the input image by dividing the region according to the color difference; (2) color rendering, which encodes the scene as voxel embeddings and outputs the rendered color and SDF value of a given pixel through a KAN network; (3) tracking process, which optimizes the camera pose by differential rendering using RGB-D frames as input; and (4) mapping process, which reconstructs the geometry of the scene.

The system overview of this paper is shown in Fig. 1. Our system inputs consecutive RGB-D frames containing an RGB picture with $I_{i}\in R^{3}$ and a depth map with $D_{i}\in R$. We use the de-distorted model as a pinhole model, and the camera’s internal reference matrix $K\in R^{3\times 3}$ is known. Similar to traditional SLAM architectures [3–5], our system maintains two separate processes: the tracking process and the mapping process. As the front-end, the tracking process is responsible for estimating the current camera’s position. In contrast, the mapping process, as the back-end, is responsible for optimizing the global map.

When the system is booted into initialization, we first calculate the image brightness and RGB mean value for each frame in the dataset, synthesize the color difference and depth map of the RGB image region, and iteratively assign the sampled pixels through a quadtree, each iteration reduces the size by a quarter, so the iteration time is proportional to the logarithm of the required pixel point size, and the overall time complexity is kept to O(log n). Then, we create several voxels equal to the number of pixels in the first frame and run several mapping iterations to initialize the global mapping network ${RGB\_F}_{\theta }$. For the subsequent frames, the tracking process first estimates the camera pose and optimizes the exposure compensation network ${EXP\_F}_{\theta }$ by using the mapping network ${RGB\_F}_{\theta }$. Then, it sends each tracked frame to the mapping process to construct the global map. The mapping process obtains the estimated camera pose from the tracking process and assigns new voxels based on the depth map and coordinate system transformed coordinates. The new voxel-based scene is fused into the global map, and joint optimization is applied. To reduce the complexity of the optimization, we keep only a small number of keyframes, which are selected by measuring the proportion of observed voxels, and maintain long-term mapping consistency by continuously optimizing the keyframes in a fixed window.

### Spatial sampling

#### Regional color difference sampling.

**Fig 2:**
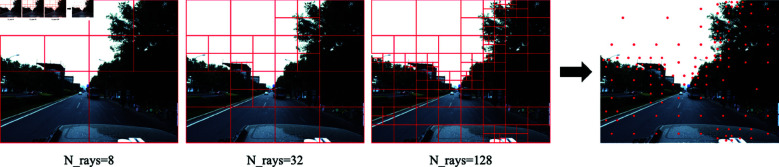
N_rays=128 sampling process.

For the sampling problem in NeRF, research generally agrees that the sampling points need to cover all the objects in the scene as much as possible (i.e., to ensure the coverage of voxels in space). In the case of a large number of objects in a large-scale scene, we need first to ensure that all objects in the scene are sampled and then sample different parts of the objects multiple times, which can improve the integrity of the reconstructed scene. The traditional NeRF [8,30,36] adopts interval sampling to select pixel points, and the light dispersed in this way may not be representative to a certain extent. Vox-Fusion [16] firstly combines the depth map and the Gumbel distribution to realize randomly sampled pixel points, but it cannot guarantee the coverage of effective voxels. [40] states that the pixels inside a region generally have gray-scale similarity, while the boundary between areas generally has gray-scale discontinuity. This means that the same object in the scene will not have a significant regional color difference. Our method is inspired by ORB-SLAM2 [4], the key is to iteratively segment the whole image using a quadtree, as in Eq 1 to calculate the color difference of the region in the segmented part, and then combine with the depth map to complete the selection of pixel points. In Fig. 2, we use a small number of sampling points for demonstration. The smaller the regional color difference, the more substantial the color consistency in the region, and the use of a small number of pixel points indicates that the region’s sampling makes the light correspond to all the sampling points cover a more comprehensive range. After applying regional color difference sampling, we use the random sampling method mentioned in [16] to complement the detailed description of different parts of the object.

$$Q=Q+\varphi \left(Quadtree\left(\min_{P\in Q}\left(\sum_{i=0}^{w_{P}\times h_{P}}{\left\|P_{i}^{c}-P_{mean}^{c}\right\|}^{2}\right)\right)\right).$$ (1)

Where Q is the queue that holds the image chunk P. Quadtree( ) is the quadtree function responsible for quadrupling a given chunk by its center point. $P_{i}^{c}$ denotes the individual pixel point RGB values in chunk P. We represent the magnitude of the color difference in each chunk by calculating the root mean square error (RMSE).

#### Voxel-based 3D sampling.

We represent the scene as a 3D voxel distribution. For a sampled pixel point obtained in Section Regional color difference sampling, we first check if it hits any voxel in the visualization by performing a ray-voxel intersection test [41]. Pixel points that do not intersect waste computational power during rendering, masking out pixel points with no hits. Because of the need to support dynamically expanding scenes, we assume that all scenes are unbounded and control the amount of data rendered by limiting the number of spatial voxels hit by a single pixel point *H*_*max*_.

### Depth and color rendering

#### Kolmogorov-Arnold network based on radial basis function.

The KAN network structure idea comes from the Kolmogorov-Arnold [25] representation theorem, which states that every multivariate continuous function defined over a bounded domain can be represented as a finite combination of multiple univariate continuous functions φ connected by an addition operation. When we consider the relationship between spatial coordinates and the corresponding color and depth values as a high-dimensional function mapping, for machine learning, the process can be reduced to learning one-dimensional functions with polynomial weight changes as in Eq 2:

$$KAN(x)=\left(\varphi_{L-1}\circ \varphi_{L-1}\circ \cdots \circ \varphi_{1}\circ \varphi_{0}\right)x.$$ (2)

MLP networks use global activation (e.g., ReLU, Tanh, SiLU), and any local variations may propagate uncontrollably and continuously, leading to catastrophic forgetting problems [42] in large-scene NeRFs. KAN networks take advantage of the localization of the univariate functions, and subsequent samples will only affect the coefficients of some nearby univariate functions, guaranteeing the validity of the training of the antecedent data. In this paper, the Gaussian function [43] in the radial basis function is used to parameterize each univariate function. At the same time, a residual-like connection is used to connect a basis function Eq 4 so that the activation function Eq 3 is the sum of the basis function and the Gaussian function. The coefficients ω of each Gaussian function are gradually learned so that the final approximation of the mapping function between the spatial coordinates and the color and depth values is achieved:

$$\varphi (x)=b(x)+\omega \cdot e^{-{\left(\frac{x-\mu }{\sigma }\right)}^{2}},$$ (3)

$$b(x)=\frac{x}{1+e^{-x}}.$$ (4)

Where μ is the mean and σ is the standard deviation, determined by the set grid spacing.

#### Network model.

**Fig 3:**
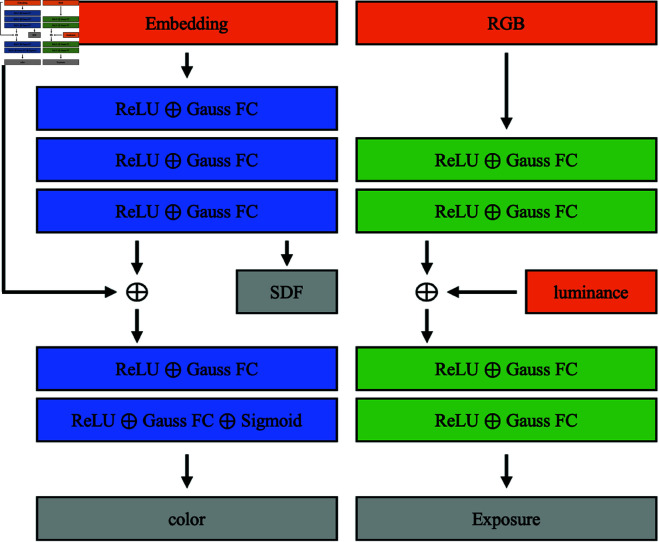
Network architecture.

Our network results are shown in Fig 3. Unlike the traditional NeRF [8], we use the surface class expression [16,18,44]. By inputting a sampling point, the symbolic distance function SDF outputs the distance to the nearest surface in space to that point, and the surface class method determines that the closer the sampling point to the surface, the higher the color contribution. The key to our method is the use of a combination of 3D coordinates and voxel embedding as described in the following Eq 5:

$$\left(C_{i},S_{i}\right)={RGBD\_F}_{\theta }\left(\alpha \left(t,T,L\right)\cdot TriLerp\left(\xi_{i}T_{i}p_{i},Vex(e)\right)\right),$$ (5)

$$\beta_{i}={Exp\_F}_{\theta }\left(\left(\overset{-}{R},\overset{-}{G},\overset{-}{B}\right),\left(max\left(L_{i}\right),\overset{-}{L_{i}}\right)\right),$$ (6)

$$L_{i}=C_{i}^{gt}\cdot Y,$$ (7)

$$w_{i}=\sigma \left(\frac{S_{i}}{tr}\right)\cdot \sigma \left(-\frac{S_{i}}{tr}\right),$$ (8)

$$C=\frac{1}{\sum_{j=0}^{N-1}w_{j}}\sum_{i=0}^{N-1}w_{j}\cdot \beta_{i}\cdot c_{j},$$ (9)

$$D=\frac{1}{\sum_{j=0}^{N-1}w_{i}}\sum_{j=0}^{N-1}w_{j}\cdot d_{j}.$$ (10)

Where Vex( ) is a function that combines voxel embedding e and 3D coordinates, *T*_*i*_ is the current camera pose, TriLerp(,) is a trilinear interpolation function, ξ is the optimization coefficient of the camera pose by trilinear interpolating 3D coordinate voxel embeddings. α(t,T,L) is the frequency regularization [22,23] of the current round, and ${RGB\_F}_{\theta }$ is the implicit network with the training parameter θ. *C*_*i*_ is the predicted RGB value of 3D points obtained by neural network, and similarly *S*_*i*_ is the predicted SDF value. y is the RGB three-channel weight value, which is generally set to [0.299, 0.587, 0.114], ${EXP\_F}_{\theta }$ is the exposure compensation network with a trainable parameter θ, and $C_{i}^{gt}$ is the real value captured by the camera. σ( ) is the sigmoid function and tr is a predefined truncation distance.

#### Optimization.

To supervise network learning, we apply four different loss functions to the sampling points p: RGB loss, depth loss, SDF loss, and VIT loss. The RGB and depth losses as shown in Eq 11 are the absolute differences between the rendered and authentic images:

$${ℒ}_{RGB}=\frac{1}{p}\sum_{i=0}^{p}\left\|C_{i}-C_{i}^{gt}\right\|,\;{ℒ}_{Depth}=\frac{1}{p}\sum_{i=0}^{p}\left\|D_{i}-D_{i}^{gt}\right\|.$$ (11)

Where *C*_*i*_ and *D*_*i*_ are the RGB and depth values of the ith rendering result, and $C_{i}^{gt}$ and $D_{i}^{gt}$ are the corresponding camera shot absolute values. Meanwhile, we apply SDF loss as shown in Eq 12 to force the network to learn the exact surface representation within the surface truncation region:

$${ℒ}_{SDF}=\frac{1}{p}\sum_{p\in P}\frac{1}{S_{p}^{tr}}\sum_{s\in S_{p}^{tr}}{\left(D_{s}-D_{s}^{gt}\right)}^{2},$$ (12)

We use DINO-VIT [45], a self-supervised vision converter trained on the ImageNet dataset, to achieve semantic consistency, and the VIT architecture captures semantic appearance after self-supervised pre-training. Unlike DietNeRF [30], which utilizes CLIP-VIT [46] and employs its projected image embeddings as features, we extract CLS tokens directly from the output of DINO-VIT, which is a more straightforward approach because CLS tokens can be used as a representation of the entire image. We compute the distance between the extracted features as in Eq 13:

$${ℒ}_{CLS}={\left\|f_{vit}(A)-f_{vit}(B)\right\|}^{2},$$ (13)

where $f_{vit}(\;)$ refers to the extracted CLS token. A and B are from the reference view and the view predicted by the neural network, respectively. Finally, we get the complete loss function as in Eq 14:

$${ℒ}_{ALL}=\lambda_{1}\cdot {ℒ}_{RGB}+\lambda_{2}\cdot {ℒ}_{Depth}+\lambda_{3}\cdot {ℒ}_{SDF}+\lambda_{4}\cdot {ℒ}_{CLS},$$ (14)

$\lambda_{1}$, $\lambda_{2}$, $\lambda_{3}$, and $\lambda_{4}$ are weighting factors for each loss.

### Tracking

We optimize the exposure compensation and camera position while keeping the implicit network and voxel 3D coordinate embedding constant during the tracking process. For new view frames entering the tracking process, we assume as in [16,17] that the new view frame is in a stationary model due to the short time between the new view frame and the last tracked frame. Therefore, we use the pose of the previously tracked frame to initialize the pose of the new frame. We follow the procedure described in Section Spatial sampling, Section Depth and color rendering for each frame that enters the tracking process, perform the operations of sampling and rendering for the current frame and iterate multiple times. During the iteration process, we use frequency regularization as shown in Eq 15 depending on the round of iteration, and it was demonstrated in [23] that the introduction of frequency regularization balances the high-frequency details with the generalization ability of the model:

$$\alpha_{i}\left(t,T,L\right)=\left\{ \begin{array}{c} 1\;\;\;\;\;\;if\;i\leq \frac{t\cdot L}{T}+3\\ \frac{t\cdot L}{T}-\left\lfloor \frac{t\cdot L}{T}\right\rfloor \;\;\;\;\;\;if\;\frac{t\cdot L}{T}+3<i\leq \frac{t\cdot L}{T}+6\\ 0\;\;\;\;\;\;if\;i<\frac{t\cdot L}{T}+6 \end{array}\right.$$ (15)

Where t is the current iteration round, T is the total number of iteration rounds, and L is the embedding length. Finally, the frame pose and associated embedding are updated based on iterative backpropagation. We keep the number of local map voxels *N*_*h*_ per frame hit in the tracking process, and this parameter can be directly used to select keyframes in the mapping process.

### Mapping

#### Keyframe selection.

In SLAM systems, selecting keyframes is the key to ensuring long-term map consistency and preventing catastrophic forgetting. Traditional SLAM based on feature points and so on [3–5,47,48] follow two principles in the selection of keyframes: the number of matching points between the current frame and the local map is less than a certain threshold; the current frame is far away from the previous keyframe in time and distance.

We follow the above idea and propose a way to insert keyframes based on voxel hit rate and time difference. Specifically, we take the voxel hit number *N*_*h*_ obtained in Section Tracking, and based on the number of sampling points *N*_*s*_ in each frame, we can derive the hit ratio $ratio=N_{h}/N_{s}$, and insert a new keyframe if the ratio is smaller than a certain threshold; when the camera’s trajectory presents a closed-loop state, we will keep observing a known scene model, and the voxel hit ratio will always be satisfied. This loses the information contained in subsequent view frames. We, therefore, enforce a maximum time interval, and a new keyframe is created when the time interval between the current frame and the previous keyframe satisfies the condition.

At the beginning of the system startup, there was a large error in the mapping process due to the small number of keyframes, and keyframes needed to be inserted as soon as possible. Therefore, we use a high voxel hit rate and short time interval to increase the keyframes quickly, and in all our experiments, the voxel hit rate gradually and linearly decreases from 0.3 to 0.1 in the initialization, and the time interval linearly increases from 5 to 10 frames.

#### Mapping and pose updates.

The mapping process obtains the RGB-D frames of the tracking process and fuses them into the existing scene by co-optimizing the scene geometry and camera pose. In the process of joint optimization, we arrange the size of the optimization window and the way of selecting it, considering that using too many keyframes to compose the optimization window will reduce the efficiency of the system operation. The camera trajectories of the indoor dataset and the NeRF dataset [49] are centripetal or centrifugal and highly overlapping, so random selection of keyframes in the co-optimization process ensures the richness of viewpoints. The camera trajectory of the urban road dataset can be considered to be unidirectional without considering the complexities such as loopbacks, and the voxels that can observe the current frame must be the previous keyframes, and the optimization window is chosen to be the penultimate keyframes of a fixed size.

Similar to the tracking process, the process described in Section Spatial sampling, Section Depth and color rendering is followed, where the current frame is subjected to the operations of sampling and rendering with several iterations. Finally, the keyframe pose, the associated embedding, the exposure compensation model, and the scene implicit network are updated based on iterative backpropagation.

## Results

We evaluate our SLAM framework on real datasets from different scenarios. We also conduct several comprehensive ablation studies to support our design.

### Experimental configuration

#### Datasets.

Our experiments use three different datasets: (1) The Kitti dataset [19], which contains four different scenes. (2) Apollo dataset [21] contains street scene data of 5 road segments. (3) Replica dataset [49]. Since similar studies cannot completely reconstruct large outdoor scene datasets, we use the Kitti and Apollo datasets to compare reconstruction stability with similar studies and the Replica dataset to compare position estimation accuracy and reconstruction quality with similar studies. Therefore, these datasets cover indoor and outdoor scenes and are well suited to study our proposed system.

#### Assessment of indicators.

We evaluate the scene geometry in both 2D and 3D, respectively. For the 2D measure, we evaluate the reconstructed imaging peak signal-to-noise ratio (PSNR), structural similarity (SSIM), and learned perceptual image block similarity (LPIPS) while comparing imaging details. For a fair comparison, we standardize the image size to 400*320. For 3D measurements, we evaluate the camera pose and trajectory using Absolute Error Trajectory (ATE) to assess the algorithm accuracy and trajectory global consistency. In addition, we evaluate the number of parameters and training time of the network structure under the same training device.

#### Realization details.

Our network architecture is divided into two parts, where the input to the prediction part is a 16-D feature embedding, and these features are processed by two Gauss FC layers, each set with 32 hidden units. The SDF header outputs a scalar SDF value and a 16-D feature vector. The Color header sets up two Gauss FC layers, each set with 32 hidden units, and outputs RGB values in the range of [0,1] using the sigmoid function to output RGB values in the range [0,1]. The input to the exposure compensation part is a 3-D mean value of the three color components of RGB in a white point set consisting of some pixels and a 2-D set consisting of the maximum and mean values of the luminance components of the picture. Two Gauss FC layers process the mean values, each set with 16 hidden units. The Exp header sets two Gauss FC layers, each set with 16 hidden units, and finally, outputs Exp values in the range [0,1] using the sigmoid function. For all scenes, we use a voxel size of 0.2m, and the white point set consists of the first 90% of the luminance component of each pixel.

We ran the SLAM system on Ubuntu 18.0.4 with a 2.8 GHz Intel(R) Xeon(R) Platinum 8362 CPU and an NVIDIA RTX 3090 GPU. In all our experiments, the loss function weights are uniformly set to $\lambda_{RGB}$=2.0, $\lambda_{Depth}$=1.0, and $\lambda_{VIT}$=0.1. For small-scale synthetic datasets, we choose N_rays=2048; for large outdoor scene datasets (Kitti [19] and Apollo [21]), we use N_rays=4096 to guarantee the hits.

#### Selection of basis functions.

There are many choices of basis functions for the KAN network, the common ones are the BSplines basis function, Fourier function, GRBF basis function, and Chebyshev function, etc. We replace the various basis functions into the network structure described in Section Realization details and select the optimal basis function by comparing the number of parameters, training speed, and other factors. The Table 1 shows that our selected basis functions can win with excellent performance compared to similar basis functions and only lack training speed compared to traditional MLP networks. Therefore, the GRBF basis function is the most suitable as the basis function for the KAN network.

**Table 1:** The performance of various basis functions in the structure of the KAN network.

Models	Layer Params↓	Train Time / Frame (s)↓	PSNR↑	SSIM↑	LPIPS↓
Traditional-MLP	76394	3.6	29.85	0.91	0.10
BSplines-KAN	62430	13.9	33.75	0.92	0.05
Fourier-KAN	40288	6.4	33.11	0.91	0.07
FCN-KAN	39994	23.2	30.97	0.89	0.12
Chebyshev-KAN	33916	10.3	29.81	0.88	0.15
GRBF-KAN(Ours)	28916	4.9	33.81	0.92	0.05

### Reconstructing the scene

#### Reconstruction of 2D assessment.

In Table 2, we compare the results of our method in the three datasets with other methods, with some of SplaTAM’s results coming from their paper [50] and the rest of the data coming from their officially released code runs [16,17]. We selected the two most representative sequences in each dataset as the data supporting our experiments:

**Table 2:** Reconstructed imaging PSNR, SSIM, and LPIPS.

Methods	Metrics	Kitti-01	Kitti-04	Apollo- record001	Apollo- record002	Room-0	Office-0	Ave
Nice-SLAM [17]	PSNR↑	✗	✗	✗	✗	32.33	34.6	33.47
	SSIM↑	✗	✗	✗	✗	0.84	0.87	0.86
	LPIPS↓	✗	✗	✗	✗	0.13	0.11	0.12
Vox-Fusion [16]	PSNR↑	26.87	29.9	✗	✗	33.26	35.57	31.40
	SSIM↑	0.39	0.49	✗	✗	0.88	0.9	0.67
	LPIPS↓	0.81	0.55	✗	✗	0.06	0.06	0.37
SplaTAM [50]	PSNR↑	30.15	29.27	30.79	27.58	32.86	38.26	31.49
	SSIM↑	0.53	0.42	0.56	0.4	0.98	0.98	0.65
	LPIPS↓	0.47	0.5	0.47	0.62	0.07	0.09	0.37
Ours	PSNR↑	29.74	31.13	31.73	28.3	33.81	34.42	31.52
	SSIM↑	0.52	0.55	0.63	0.47	0.92	0.84	0.66
	LPIPS↓	0.44	0.39	0.31	0.57	0.05	0.11	0.31

All results in the table are the mean values of images rendered at intervals of 25 frames, and the mean values of methods that did not complete all datasets are not counted in the comparison. Our method outperforms others in most datasets.

**Fig 4:**
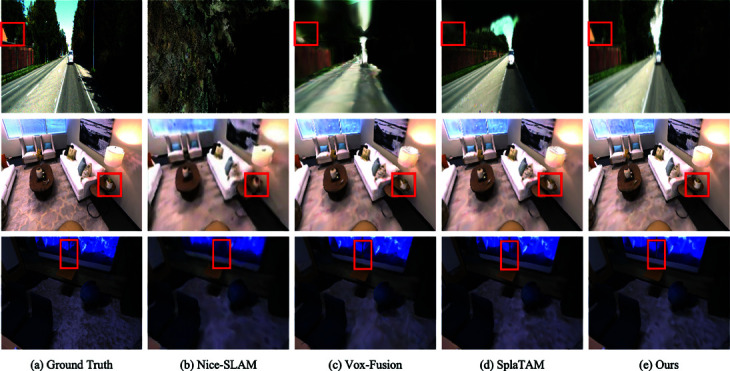
Imaging detail comparison. Our methods have obtained better results in detail rendering.

On Replica [49], our method obtains comparable capability in Vox-Fusion [16] and Nice-SLAM [17], which are also implicit representations, but the performance on the office-0 dataset is not satisfactory. We hypothesize that the poor performance is due to the miniature scene space of the office-0 dataset, the object’s surface is too close to the camera, and we sampled less of the whole space because we limited the number of spatial voxels hit by a single pixel point. We analyze that the Replica dataset artificially removes the factor of light variations present in the real environment, our method does not have a significant improvement in imaging results with the implicit expression without exposure compensation. While comparing SplaTAM [50] based on the 3DGS display representation [51,52] is slightly inferior in SSIM, we believe that the implicit representation learns the global mapping process slower than the display representation in the early stage of the operation resulting in a gap in calculating the mean value of the imaging results. The last two rows in Fig 4 compare frame 1700 of the room0 dataset with frame 1100 of the office0 dataset. From the comparison of the rendering of the potted plant on the table and the dividing line in the middle of the drawing board in the figure, we can see that our method obtains better imaging details when the learning process tends to stabilize.

In the two datasets of the outdoor road scene, Vox-Fusion [16] shows a huge deviation from frame 97 until a complete loss of localization in the Apollo record001 dataset, and a similar situation occurs in Nice-SLAM [17]. We analyze that [17] and [16] may be unable to track the camera pose correctly due to catastrophic forgetting during MLP-based implicit representation due to oversized scene representation. Our approach ensures that no catastrophic forgetting occurs due to the unique localization of the KAN network, and better results are obtained on both outdoor road datasets.

#### Reconstruction of 3D assessment.

**Fig 5:**
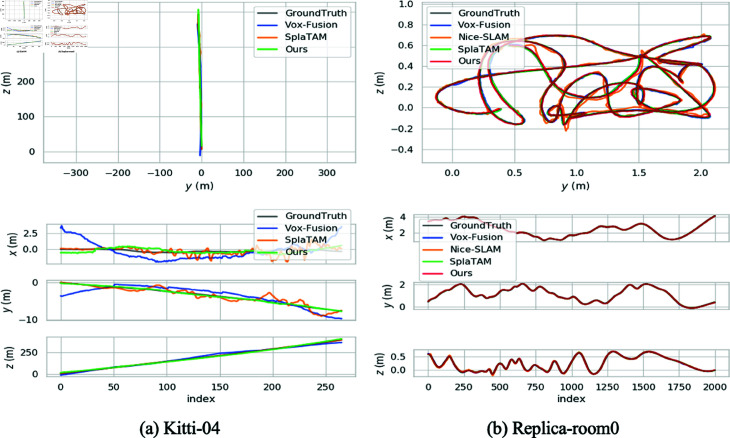
EVO tool to visualize trajectories. Our method is more stable and close to Ground Truth.

In Table 3, we compare the camera pose estimation results of our method with other methods. All results are derived from official published code runs. On Replica [49], SplaTAM [50] accomplishes the attitude estimation with a clear advantage, but as can be seen in Fig 5, Our method generates trajectories that are as close to the actual values as SplaTAM. As for the three implicitly expressed methods, our method controls the trajectory error accuracy from the centimeter level to within the millimeter level, and the average error is reduced by 46.3% from 1.6cm to 0.86 cm in Nice-SLAM [17], which is a 71.9% reduction comparing to Vox-Fusion [16].

**Table 3:** Camera pose estimation results (ATE RMSE [cm]) for each model on different datasets were compared with conventional vision SLAM.

Methods	Metrics	Kitti-01	Kitti-04	Apollo- record001	Apollo- record002	Room-0	Office-0	Ave
Nice-SLAM [17]	RMSE↓	✗	✗	✗	✗	1.93	1.27	1.6
	Mean↓	✗	✗	✗	✗	1.67	1.04	1.36
	Median↓	✗	✗	✗	✗	1.43	0.83	1.13
Vox-Fusion [16]	RMSE↓	13.57	11.15	✗	✗	0.88	5.26	7.72
	Mean↓	10.38	9.95	✗	✗	0.74	2.60	5.92
	Median↓	8.75	8.17	✗	✗	0.63	1.72	4.82
SplaTAM [50]	RMSE↓	9.53	9.88	13.74	16.39	0.31	0.47	8.39
	Mean↓	8.44	9.52	11.96	13.73	0.29	0.41	7.39
	Median↓	7.86	7.18	10.83	12.57	0.28	0.39	6.52
Ours	RMSE↓	10.10	8.51	12.37	13.28	0.73	0.99	7.66
	Mean↓	8.37	8.08	9.77	11.56	0.61	0.74	6.52
	Median↓	7.95	7.90	7.87	10.83	0.59	0.70	5.97
ORB-SLAM3 [5]	RMSE↓	6.40	6.16	5.29	7.92	0.62	0.46	4.48
	Mean↓	5.00	5.08	3.89	6.40	0.58	0.42	3.56
	Median↓	4.64	4.37	3.86	6.22	0.59	0.39	3.35

The mean values of methods that did not complete all datasets are not counted in the comparison. Our process is optimal in machine learning classification for the outdoor road dataset, and the indoor synthetic dataset is close to SplaTAM, with a gap to ORB-SLAM3.

On the outdoor road scene, Nice-SLAM [17] failed to run completely for both datasets, Vox-Fusion [16] failed to run completely for the Apollo dataset [21], and the Kitti dataset [19] was able to run partially. By looking at the images rendered by both methods before the crash, we learned that both methods had significant artifacts and debris, which in turn caused the crash by being unable to restore the camera’s correct bit position through the implicit scene. Since both methods estimated partial trajectories before crashing, we collect the data in Table 4. In contrast, our method successfully tracks the camera on partial sequences of both datasets and provides more competitive performance regarding trajectory error. The results of this dataset show that we can also restore the trajectory of the camera motion more accurately for data from outdoor sparse views, which is impossible with other MLP-based NeRF-based SLAMs.

**Table 4:** Partial running attitude estimation trajectory (RMSE[cm]).

Methods	Kitti-01	Kitti-04	Apollo- record001	Apollo- record002
Nice-SLAM [17]	17.15	15.84	9.35	23.6
Vox-Fusion [16]	13.57	11.15	13.28	16.81
Ours	6.40	6.16	5.29	7.92

Since the core objective of NeRF is dense scene reconstruction and rendering, pose estimation is usually used as an auxiliary task. Its pose optimization relies on an implicit representation of the scene reconstruction, which may introduce cumulative errors with lower accuracy than traditional methods that directly optimize the reprojection error [53], so we also compare our SLAM with the SOTA SLAM method [5] to recognize the gap. The results show that our method reduces the accuracy gap to the centimeter level for outdoor scenes and keeps the indoor gap to the millimeter level when comparing the two methods with the exact implicit neural radiation field representation. Comparing the 3DGS methods flagging and slightly better in the resultant mean, our process is closest to the ORB-SLAM3 [5].

### Ablation experiment

We utilize the code of the original NeRF, retain its modules for image rendering, and add Tracking and Mapping threads, bit pose estimation optimization, and depth maps to it, and finally construct NeRF-SLAM, which will be used as the substrate for the ablation experiments.

#### Network forgetting.

**Fig 6:**
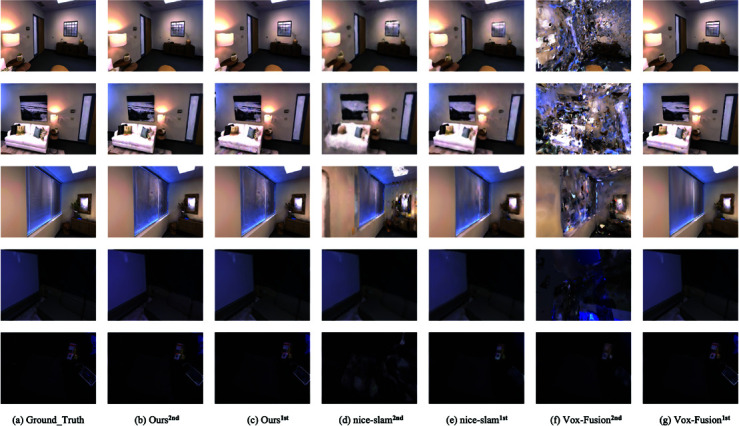
Comparison of secondary reconstruction renderings. The label 1st denotes the rendering obtained with normal execution of the tracking and mapping threads, and 2nd denotes the second reconstruction rendering after fixing the network parameters. To make the problem of network forgetting more apparent, the image frames we compare are in the first 40% frames of the whole dataset. Our approach is free of network forgetting.

In Table 5, we compare the two methods of using MLP network structure as scene representation. In the dataset of four indoor scenes, there are apparent trajectory deviations in the study using MLP network. Through Fig. 6, we can see that Nice-SLAM has blurring, lines appearing wavy and artifacts in the second rendering, and the overall quality is significantly reduced compared to the first rendering, which indicates that there is a network forgetting problem. In contrast, Vox-Fusion has significant artifacts and serious network forgetting problem in the second rendering. The results of the second rendering of both are consistent with the trajectory offsets in the table, and we conjecture that Nice-SLAM mitigates the MLP network forgetting problem to a certain extent due to the use of hierarchical scene representation based on feature grids. Our method’s frame comparison at secondary rendering appears closer to reality for the first time, and we conjecture that this is a result of the continuous refinement of the scene in subsequent frames during training and the absence of the network forgetting problem.

**Table 5:** Results of the quadratic position estimation (ATE RMSE [cm]) for each model in the replica dataset. Our method minimizes the error in the quadratic position estimation without significant bias.

Methods	Metrics	Room-0	Room-1	Office-0	Office-1	Ave
Nice-SLAM [17]	RMSE↓	2.34	10.65	1.13	84.51	24.66
	Mean↓	1.78	5.87	0.85	75.91	21.10
	Median↓	1.31	2.79	0.66	69.74	18.63
Vox-Fusion [16]	RMSE↓	94.88	3.42	76.99	3.60	44.72
	Mean↓	87.73	2.86	72.72	3.31	41.66
	Median↓	87.02	2.42	70.95	3.20	40.9
NeRF-SLAM	RMSE↓	186.33	72.65	137.01	175.54	142.88
	Mean↓	152.85	69.43	113.64	149.80	121.43
	Median↓	134.34	68.29	107.93	131.33	110.47
Ours	RMSE↓	1.33	1.88	1.09	2.83	1.78
	Mean↓	1.16	1.71	0.97	2.45	1.57
	Median↓	1.02	1.52	0.81	2.07	1.36

We revalidate the dataset by fixing the network parameters after the scene reconstruction (i.e., re-estimating the bitmap and rendering the image data from the same dataset after completing the tracking and image-building threads) and apply the same process to similar studies.

#### Region sampling and VIT loss.

**Fig 7:**
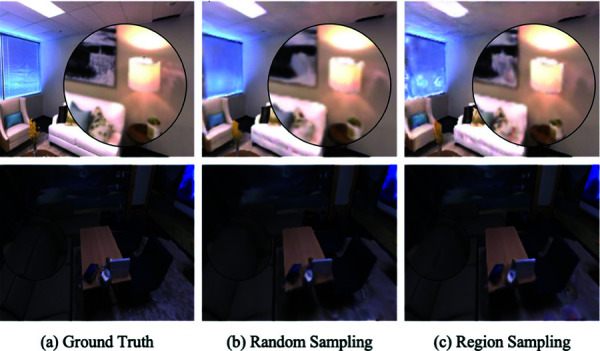
Compare and contrast regional chromatic aberration sampling with random sampling. Using regional color difference sampling gives better detail in the early stages of mapping.

We modify the NeRF-SLAM network structure while maintaining its sampling strategy and loss function, respectively, and test the strengths and weaknesses of the system on two datasets, room0 and office0. In Table 6, in the case of using VIT loss, we conjecture that the camera trajectory error is slightly degraded due to the slight change in bit position forced by the strict loss. Still, the image quality is improved to some extent. Using region sampling instead of random sampling, the camera trajectory error is slightly improved, but the image quality is substantially improved. As can be seen in Fig. 7, region sampling yields better detail at the beginning of the mapping process.

**Table 6:** Ablation experiments for each module were performed in the replica dataset.

Methods	Metrics	Room-0	Office-0	Ave
NeRF-SLAM	ATE (cm)↓	9.31	10.04	9.68
	PSNR↑	27.95	30.17	29.06
Regional-only	ATE (cm)↓	9.26	9.88	9.57
	PSNR↑	30.02	32.45	31.24
VIT-only	ATE (cm)↓	9.86	10.92	10.39
	PSNR↑	29.79	31.50	30.65

#### Exposure compensation.

**Fig 8:**
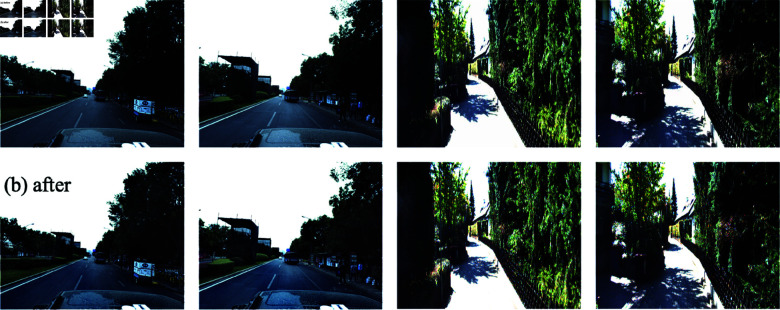
Exposure compensation conversion chart.

Since the indoor scene dataset is less affected by the lighting problem, we use the Kitti dataset to validate this ablation experiment in Fig 8. Meanwhile, using the MLP network structure of NeRF-SLAM cannot stabilize the reconstructed dataset; we compare the RSNeRF with exposure compensation removed with the full RSNeRF. From the Table 7, it can be seen that the removal of exposure compensation camera trajectory error and image quality at the same time show a significant decrease, which shows that the light transformation affects the quality of reconstruction for outdoor scenes.

**Table 7:** Ablation experiment for exposure compensation.

Methods	Metrics	Room-0	Office-0	Ave
Without Exp	ATE (cm)↓	11.93	9.00	10.47
	PSNR↑	28.41	30.38	29.40
Ours	ATE (cm)↓	10.10	8.51	9.31
	PSNR↑	29.74	31.13	30.44

### Computational overhead and efficiency

In Table 8, we compare our running time and the number of network parameters with others. We uniformly set the number of tracking process iterations to 50 and the number of mapping process iterations to 30. SplaTAM [50] obtains the best accuracy because of the huge time overhead, while Nice-SLAM [17] obtains poor trajectory error accuracy because of the faster computation time. Our method balances time, space overhead, and accuracy better.

**Table 8:** RTX 3090 GPU running Replica room0 correlation data.

Methods	Layer Params↓	Train Time / Frame (s)↓	ATE RMSE(cm)↓
Nice-SLAM [17]	58956	2.7	1.928
Vox-Fusion [16]	141188	3.4	0.877
SplaTAM [50]		12.1	0.312
Ours	28916	4.9	0.728
The training time for each frame is total time=training time/number of samples.			

## Discussion

We propose an implicitly expressed SLAM system based on KAN networks. Our method employs region sampling, which improves the hit rate of sampling and helps sample objects in space more efficiently. In addition, we apply the advantages of the KAN network and combine it with various regularizations to realize the reconstruction of outdoor road scenes, which cannot be achieved by other implicit neural networks, and experimentally prove its effectiveness, which improves the accuracy of the camera pose estimation and the robustness of the reconstruction to a certain extent, and has a wide range of prospects for real-world applications. Skin can help vehicle sensing in the field of automated driving by accurate pose estimation with RSKAN can help vehicle perception in the field of autonomous driving with precise pose estimation and highly robust reconstruction. It can also help intelligent robots construct maps, plan paths, avoid obstacles, and a series of other tasks, promoting the NeRF-related SLAM technology to the ground. At present, our research has significant bottlenecks in dynamic object modeling, cross-scene generalization, and real-time performance. Erroneous introduction of dynamic objects quickly leads to trajectory tracking failure or affects reconstruction with artifacts, and the lack of generalization and real-time performance affects the application and deployment of our research in real scenes. Still, including the KAN network provides some help for more complex and efficient NeRF-based SLAM in the future.

## Supporting information

S1 Text Project source codes. We publish part of our project code and dataset profiles at GitHub.
(DOCX)
