# Supplementary material for: Region sampling NeRF-SLAM based on Kolmogorov-Arnold network
Source: PLoS One. 2025 May 27;20(5):e0325024. doi: 10.1371/journal.pone.0325024 (PMC12111630; doi:10.1371/journal.pone.0325024)
Supplement: S1 Text — We publish part of our project code and dataset profiles at GitHub. (DOCX) [file pone.0325024.s001.docx]

Region Sampling NeRF-SLAM based on Kolmogorov-Arnold Network: Supporting Information

Zhanrong Li. Jiajie Han Chao Jiang Haosheng Su

**Appendices**

1. S1 GitHub repository.
